# Interactions between the hippocampus, prefrontal cortex, and amygdala support complex learning and memory

**DOI:** 10.12688/f1000research.19317.1

**Published:** 2019-07-31

**Authors:** Ersin Yavas, Sarah Gonzalez, Michael S. Fanselow

**Affiliations:** 1 Psychology, University of California, Los Angeles, Los Angeles, CA, 90095, USA; 2 Psychiatry and Biobehavioral Sciences, University of California, Los Angeles, Los Angeles, CA, 90095, USA

**Keywords:** fear, learning, amygdala, hippocampus, prefrontal cortex

## Abstract

One of the guiding principles of memory research in the preceding decades is multiple memory systems theory, which links specific task demands to specific anatomical structures and circuits that are thought to act orthogonally with respect to each other. We argue that this view does not capture the nature of learning and memory when any degree of complexity is introduced. In most situations, memory requires interactions between these circuits and they can act in a facilitative manner to generate adaptive behavior.

## Introduction

There is no doubt that most animal species depend on the ability to learn for survival. Learning and the behaviors through which learning is expressed require interactions between large-scale brain networks. In mammals, simple forms of learning, such as when X perfectly predicts Y, often can be handled by subcortical networks. Take, for example, basic Pavlovian fear conditioning in which a tone signals aversive shock 100% of the time and shock never occurs in the absence of tone. In this case, projections from auditory thalamus to amygdala to midbrain periaqueductal gray are sufficient for learning ^1^. However, as learning becomes more complex, memory and recall depend on interactions between the cortex and hippocampus and through those interactions these structures modulate the subcortical circuits that support simple learning. Healthy behavior depends on these interactions and abnormal structure and function in these regions are associated with a variety of neuropathology and psychopathology, including anxiety disorders, schizophrenia, and Alzheimer’s dementia ^2– 4^. Although the roles of both the hippocampus and frontal cortex in regulating complex behavior have been studied extensively, much has been learned recently about how the interactions between these regions impact learning and memory-related functions.

## When are hippocampal–cortical interactions necessary for memory?

Traditionally, the relationship between brain structures and forms of memory has been conceptualized under a multiple memory systems taxonomy, in which specific structures are linked to specific classes of learned behavior ^5^. In this taxonomy, the hippocampus is associated with episodic memory, the striatum with skill learning, the neocortex with perceptual learning, and the amygdala with emotional memory. Although this taxonomy has been a major impetus to memory research and spurred considerable advancement in our understanding of learning, it fosters the idea that there is a simple one-to-one correspondence between a single structure and a type of memory and that these separate systems act in an orthogonal manner ^6^. Such a view fails to capture the fact that even modestly complex memory emerges from a network of interactions between these structures.

## Complex versus simple stimuli

Above we described fear conditioning, in which a tone perfectly predicts shock, as an example of simple learning that can be carried out by a subcortical circuit. However, when experiencing fear conditioning, the subject simultaneously learns that the context or place where this learning occurs is also associated with shock, enabling that context to trigger fear and avoidance ^7^. Contexts are complex stimuli made of many features, all of which may lack the salience of the sudden onset of a simple auditory stimulus such as a tone ^8^. The context is also less precisely paired with the stimulus that reinforces learning (in this case, the shock). This modest increase in complexity cannot be handled by the simple subcortical circuit and instead requires an orchestrated dance between hippocampus, prefrontal cortex, and amygdala ^9^.

Consistent with their role in spatial memory, dorsal hippocampal neurons appear to encode the details of the context supporting recognition of the trained environment. Two groups of rats were placed in a novel chamber for 5 min where they received shock. One group received the shock in a manner that promoted fear learning (at the end of the session) and the other in a manner that did not promote fear learning (at the beginning of the session). When these rats were returned to the same chamber neurons that were active during the initial exposure tended to reactivate during the second exposure indicating that the hippocampus recognized the chamber as familiar. Interestingly, the same number of neurons reactivated in both groups even though they showed large differences in fear expression, suggesting that hippocampus is not essential for the coding of affective features of the context ^9^. On the other hand, basolateral amygdala neurons reactivate only if the animal has learned fear of the context, indicating that the amygdala is involved in the affective component of memory.

These two sources of information appear to come together in the prelimbic portion of the prefrontal cortex as activity in that region reflects a sum of that found in hippocampus and amygdala ^9^. In support of this, coordinated 4-Hz rhythmic activity between the prelimbic and amygdala regions accurately predicts freezing, a behavioral manifestation of fear ^10^. Coordinated oscillatory activity between hippocampus and prelimbic regions may be the method by which the hippocampus provides spatial information to the prelimbic cortex ^11^.

The dynamics between the hippocampus, cortex, and amygdala depend on the length of memory retention ^7, 12^. Shortly after learning, the memory is dependent on the hippocampus and there is more hippocampal activity during memory retrieval ^13^. However, at later time points (weeks to months), the prefrontal cortex takes on the larger role. A recent study indicated that the development of this prefrontal representation of contextual memory is dependent on inputs from the entorhinal cortex, hippocampus, and basolateral amygdala during learning ^14^. The changes that occur over these long time periods are referred to as systems consolidation.

This coordination of regions during systems consolidation may occur off-line during the replay of events that occurs during sleep ^15^. The integration of spatial information with emotional memory may also occur during non-REM sleep when there is a coordinated replay of the amygdala and hippocampal activity that occurred during learning ^16^.

Interestingly, the prefrontal cortex can compensate for loss of hippocampal function to a certain degree, but the resulting memories differ from those formed using an intact hippocampus. Following damage to the dorsal hippocampus, contextual fear memories can be formed after extensive training but these memories lack the permanence of those formed using an intact hippocampus ^17, 18^. This compensation depends on communication between the infralimbic and prelimbic regions of the prefrontal cortex. Damage to and disconnection between these two adjacent prefrontal regions abolish contextual fear learning entirely when combined with dorsal hippocampal damage ^18^.

## Certain versus uncertain predictors

While learning that an auditory stimulus consistently signals shock can be accomplished by a subcortical circuit, this changes when the relationship between the tone and shock becomes ambiguous. The simplest and most translationally relevant example of this is fear extinction, in which a stimulus that previously received consistent reinforcement is now presented without reinforcement. Because of this inconsistent reinforcement, the animal treats the tone as ambiguous and uses context to resolve the decision of whether or not to respond ^19^. This decision also relies on interactions between hippocampus, prefrontal cortex, and amygdala. When communication between the ventral hippocampus and prelimbic cortex or between the ventral hippocampus and amygdala is prevented, the subject treats the tone as safe regardless of context ^20, 21^. If these connections are intact but communication between the hippocampus and infralimbic cortex or between the amygdala and infralimbic cortex is prevented, the animal treats the tone as consistently dangerous ^22, 23^. Additionally, infralimbic lesions lead to greater generalization between two contexts and simultaneously reduce the context’s ability to modulate responses to an extinguished stimulus ^18^. Recent evidence suggests that the hippocampus may help solve the problem by encoding two distinct contextual memories of the same context: one where the tone is dangerous and one where it is safe ^24^. This may allow the rapid switching that rats can accomplish between reacting to the tone as dangerous or safe on the basis of the current context ^25^.

Such a role for the prefrontal cortex in resolving ambiguity has also been shown in contextual biconditional discrimination procedures, which require subjects to use contextual information to resolve the meaning of ambiguous stimuli. In these procedures, two different stimuli are presented in two different contexts; one stimulus is predictive of an outcome in one context whereas the second stimulus is predictive of that outcome in the second context ^26– 28^. Lesions of the prelimbic cortex impair the ability of subjects to use contextual stimuli to determine which of two auditory stimuli predicts footshock, instead producing an intermediate level of fear to both stimuli ^28^.

## Conclusions

Learning is often conceptualized as occurring in distinct and independently acting brain regions. However, to navigate a complex and ever-changing environment, animals must be able to learn about complex stimuli and complex relationships between stimuli. In these instances, mammals use environmental cues to resolve the meaning of ambiguous stimuli. Rather than being dependent on any one brain region, this ability arises from a network of cortical and subcortical structures and their interactions. Here, we focused on Pavlovian conditioning because it is often thought of as a simple form of learning. However, introducing even slight complexities in this learning, such as those that occur when contexts are used as stimuli or following the change in meaning that occurs during extinction, recruits and requires long-range interactions between regions that are often thought to serve distinct domains of memory.
